# The relationship of self-reported and device-based measures of physical activity and health-related quality of life in adolescents

**DOI:** 10.1186/s12955-021-01682-3

**Published:** 2021-03-01

**Authors:** Kathrin Wunsch, Claudio R. Nigg, Susanne Weyland, Darko Jekauc, Claudia Niessner, Alexander Burchartz, Steffen Schmidt, Ann-Katrin Meyrose, Kristin Manz, Franz Baumgarten, Alexander Woll

**Affiliations:** 1 Institute of Sports and Sports Science, Karlsruhe Institute of Technology, Engler-Bunte-Ring 15, 76131 Karlsruhe, Germany; 2 Institute of Sport Science, University of Bern, Bern, Switzerland; 3 Department of Child and Adolescent Psychiatry, Psychotherapy, and Psychosomatics, University Medical Center Hamburg-Eppendorf, Hamburg, Germany; 4 Clinical Psychology, Helmut-Schmidt University, Hamburg, Germany; 5 Department of Epidemiology and Health Monitoring, Robert Koch Institute, Berlin, Germany

**Keywords:** KIDSCREEN-27, Accelerometry, Objective activity assessment, Subjective activity assessment

## Abstract

**Background:**

Physical activity (PA) has beneficial effects on health and health-related quality of life (HRQoL), which is a protective factor of illness and mortality. The purpose of this examination was to investigate if self-reported and device-based measures of PA were related to HRQoL in adolescents.

**Methods:**

Participants (*N* = 1565; 54.3% female; *M*_age_ = 14.37 years, *SD*_age_ = 1.99) were recruited from 167 sample points across Germany. Adolescents self-reported their PA, supplemented by a 1-week examination of device-based PA using accelerometry. Additionally, they completed the multidimensional KIDSCREEN-27 to assess HRQoL.

**Results:**

Results showed that self-reported PA was correlated with overall HRQoL, Physical Well-Being, Psychological Well-Being, Social Support & Peers, and School Environment, whereas device-based PA was only correlated with Physical as well as Psychological Well-Being. Further, self-reported PA significantly predicted all facets of HRQoL except for Autonomy and Parent Relations, whereas device-based PA solely heightened the amount of explained variance in the Physical Well-Being subscale.

**Conclusions:**

Findings demonstrate the importance of self-reported PA as it is related to almost all facets of HRQoL. Both measures of PA are not congruent in their relationship with HRQoL and thus implications have to be carefully considered. Future studies should investigate the direct effect of PA on HRQoL and health in a longitudinal approach to account for the causality of effects.

## Introduction

Health-related quality of life (HRQoL) is a multidimensional construct that focuses on individuals’ perceptions of their physical, psychological, and social functioning [1]. Perceived HRQoL of children and adolescents is closely related to physical and mental health status. Mental (e.g. depression, ADHD, or conduct disorder; [2]) and physical health complaints (e.g. obesity, asthma, or cardiovascular diseases; [3]) are associated with lower HRQoL in children and adolescents, which is why HRQoL measures are commonly used for diagnoses of maladaptive effects and health complaints [4]. HRQoL is also a reliable predictor of mortality and suicide and therefore a powerful construct in the operationalization of perceived health [5, 6].

Regular physical activity (PA) was repeatedly shown to elicit positive effects not only for (older) adults, but also for children and adolescents [7], including physiological (e.g. on immune functions [8], on the cardiovascular [9] as well as respiratory systems [10]) and psychological health (e.g. reduced symptoms of depression and anxiety [11, 12], improved cognitive function, or increased self-esteem [13]). Several studies assessed the relation of PA and HRQoL in children and adolescents, pointing to a positive dose–response relationship (e.g. [14]). However, evidence on the association between PA and HRQoL is limited as research has commonly focused on specific interventions or populations suffering from chronic diseases [1, 15, 16].

Whereas PA can be measured in two different ways (i.e. via self-report and via device-based, objective measures), most studies used self-reported measures of PA, which often show a deficiency of adequate reliability and validity [1, 17]. Studies have shown that retrospective self-reports often over-estimate real PA [18], especially in children and adolescents. Until today, only a few studies have used device-based PA measures to examine the association between PA and HRQoL with divergent findings. In adult samples, Hamer and Stamatakis [19] as well as Anokye and colleagues [20] used accelerometer data to explore the association between device-based PA and self-reported measures of HRQoL and well-being. Whereas Hamer and Stamatakis [19] found a robust association of self-reported PA and self-reported well-being, no such association was found for device-based measures of PA. On the contrary, Anokye and colleagues [20] found higher levels of both self-reported and device-based PA to be related to higher scores on HRQoL scales, with device-based measures showing a stronger relation to overall HRQoL than self-reported ones [20]. Studies examining this relationship in children and adolescents are scarce. Marker and colleagues summarized results on both, self-reported and device-based measures of PA in children and adolescents [21]. The authors detected 7 out of 19 studies using objective, device-based measurement of PA with pedometers or accelerometers when assessing the relationship between PA and HRQoL and criticized this lack of device-based PA measurement, as they state that self-reported measures are not an acceptable alternative since self-reported measures of PA often overestimate duration and intensity [22]. However, based on their dataset, no conclusion about the relationship between both measurement methods could be drawn. Furthermore, the possibly different nature of both PA measures needs to be accounted for when interpreting results: while self-report measures commonly examine a fixed period in a retrospective manner (i.e. participants commonly asked about their PA levels over the past four weeks, therefore aiming at measuring habitual PA levels), device-based measures assess PA levels within a pre-specified time interval (commonly one week), therefore aiming at measuring actual PA levels, which may be influenced by various external (e.g. season) and internal (i.e. mood) moderators.

Numerous studies have compared self-reported and device-based measures of PA (e.g. [23]). However, most of them have not examined the association between measures of PA and HRQoL. To the best of our knowledge, only one study compared the influence of self-reported and device-based measures of PA on HRQoL until today in the general population [20]. However, this study is representative of England and focused on adults aged between 40 and 60 years old. Unfortunately, this study operationalized its PA measures via binary variables, losing information especially in device-based measures of PA.

Taken together, studies examining the relationship between PA and HRQoL are limited in the general population and even more in children and adolescent populations. Studies assessing the relation of PA and HRQoL in children and adolescents are scarce and rarely used device-based measures of PA [19, 20], although self-reports were observed to not provide accurate estimates of the absolute amount of PA [17, 21] and are often biased by social desirability, therefore commonly representing overestimated PA levels [24]. Nevertheless, self-reports of PA are still the most frequently used. Further, from the evidence to date on PA and HRQoL, methodological quality, measurement issues, as well as inconsistencies in findings based on measurement approaches (self-report vs. device-based), have precluded conclusions from being drawn.

Hence, the current study aimed to examine the association of questionnaire-based self-reported as well as device-based measures of PA on HRQoL to examine the different impacts of habitual versus actual PA measures in children and adolescents aged 11–18 years. As especially this period is known to reveal rather large differences between boys and girls across different age groups regarding PA and HRQoL [25, 26], analyses will be controlled for concerning this peculiarity. To expand upon previous findings (e.g. [21, 27]), secondary objectives were to examine whether a self-report (habitual) measure of PA has a stronger relationship to HRQoL than a device-based measure of (actual) PA and if device-based measured PA can explain additional variance in the well-established relationship of self-reported PA and HRQoL in children and adolescents.

## Methods

### Procedure

The Ethics Committee of the Karlsruhe Institute of Technology approved the study and adolescents participated voluntarily. They provided informed assent and (parental) consent. Data were derived from the second wave of the German Health Interview and Examination Survey for Children and Adolescents (KiGGS-study) and the Motorik-Module Study (MoMo; [28]). Wave 2 Data was gathered between 2014 and 2017 (for a detailed description see [29]).

The MoMo Study is a nationwide study on physical fitness and physical activity in children and adolescents living in Germany, and part of the German Health Interview and Examination Survey for Children and Adolescents, KiGGS [30, 31]. To ensure a diverse sample of children and adolescents and to maximize representativeness, a nationwide, stratified, multi-stage sample subject recruitment in KiGGS wave 2 was carried out in two steps [32]. First, a systematic sample of 167 primary sampling points was selected from an inventory of German communities stratified according to a classification system that measures the level of urbanization and the geographic distribution [29, 31]. The sample points for KiGGS Wave 2 were the same as in the first KIGGS study wave. The probability of any community being picked was proportional to the number of inhabitants younger than 18 in that community. Second, an age-stratified sample of randomly selected children and adolescents was drawn from the official registers of residents. The final number of participants aged 4–17 years in MoMo Wave 2 was 3,708 (33.2% response).

### Participants

There were *N* = 1565 participants between 11 and 17 years of age examined at MoMo Wave 2 and included in the current sample [*M*_age_ = 14.37 years, *SD*_age_ = 1.99; female = 54.3%; 7.7% low socioeconomic status (SES), 66.1% intermediate SES, and 25.6% high SES].

### Measures

#### Demographics

Age and sex as well as the assessment of SES were based on parental information. SES was represented by a score ranging from 1 to 3 which was built from information about education, occupational status, and household net income [33], with a value of 1 labeled as low SES, 2 as medium SES and 3 as high SES.

#### Physical activity (PA)

The MoMo Physical Activity Questionnaire (MoMo-PAQ) was used to assess self-reported habitual PA in different settings (sports clubs, leisure time, and school) [26, 34]. The MoMo-PAQ consists of 28 items and measures frequency, duration, intensity, and setting of PA in a typical week. Data obtained with the MoMo-PAQ have been shown to be sufficiently reliable (test–retest reliability: *ICC* = 0.68) and valid [26]. PA in school, sports clubs, and unorganized sports was assessed by questions about duration (minutes per week) of up to four different sports in each setting and time throughout the year on which the activity took place (months per year). From this, an index reflecting total PA in minutes per average week was calculated from the sum of minutes in each setting (sports clubs + school + leisure time) [35].

In addition, device-based PA was measured by the ActiGraph GT3X+/wGT3X-BT (ActiGraph, Groningen, Netherlands). Participants were instructed to wear the accelerometer on seven consecutive days. Parents were asked to supervise the wearing of the device. A dataset was counted as being valid if participants wore the device on at least four weekdays and one weekend day and if wear-time exceeded eight hours a day [36]. As *Median*_valid_days_ was 7 throughout the sample, time spent being moderately and vigorously active was summed up over these days (irrespective of wear time days), resulting in total PA in minutes per week within the measurement period.

#### Health-related quality of life (HRQoL)

To assess adolescents’ subjective health and well-being, the KIDSCREEN-27 was used [37–39] consisting of the following five subscales (number of items in parentheses): Physical Well-Being (5), Psychological Well-Being (7), Autonomy and Parents Relation (7), Social Support and Peers (4) and School Environment (4). Items were assessed on a five-point Likert-scale ranging from *never* to *always* or from *not at all* to *extremely*. Items were reversed where necessary according to the manual to ensure that higher scores indicate better HRQoL [37]. Based on the Rasch model, a scoring algorithm was used to calculate standardized T-scores scaled with a mean of 50 and a standard deviation of 10 for each dimension to make the interpretation more applicable [38]. Items for the KIDSCREEN-10 index were derived from the 27 items, summed up, and also transformed into T-values as a global indicator of overall HRQoL [37]. The KIDSCREEN questionnaires have been repeatedly shown to generate reliable results (Cronbach’s α > 0.70) as well as to have good cross-national validity [39].

### Data analyses

All analyses were conducted using IBM SPSS 25. For descriptive analyses, means, standard deviations, and number of participants were presented for all scores of HRQoL as well as self-reported and device-based PA. Additionally, a multivariate analysis of variance (MANOVA) was conducted to examine the effects of age and sex on HRQoL, self-reported, and device-based measures of PA. Sex differences were accounted for by pairwise t-tests. Pearson correlation coefficients were used to investigate the strength and direction of the relationship between self-reported and device-based measures of PA and all subscales of HRQoL. To examine whether a self-reported measure of PA had a stronger relationship to HRQoL than a device-based measure of PA, Fisher’s z-test for dependent samples was used [40]. To examine if device-based PA explained additional variance (Δ*R*^2^) in the relationship between self-reported measures of PA and all subscales of HRQoL, multiple hierarchical linear regression analyses were conducted. Multicollinearity was controlled for, however revealing all variance inflation factors (VIF) for step 2 models to be < 1.212, therefore not violating assumptions for hierarchical regressions. To control for age and sex effects, these variables were included as additional predictors next to self-reported PA in the first step of the model, and device-based PA was added in the second step of the model. To account for multiple testing and to encounter alpha-level inflation, Bonferroni-Holm corrections [41] were applied to all p-values, and results were considered significant (marked with *) if below α < 0.05.

## Results

### Descriptive results

For self-reported PA, results of the MoMo-PAQ revealed that participants engaged in PA for 287.62 (*SD* = 199.32) minutes per week on average (equivalent to 4.44% (*SD* = 3.14%) of their daytime when a 15.15 h wake time is assumed as suggested by current literature for adolescents aged between 11 and 16 years [41]). The device-based PA measure indicated that adolescents spent 307.86 (*SD* = 134.55) minutes per week in PA (equivalent to 5.23% (*SD* = 2.31%) of their daily accelerometer wear-time). A MANOVA revealed significant effects of sex on both, self-reported (*F*(1,1053) = 11.768, *p* < 0.05, *η*_p_^2^ = 0.011) and device-based PA (*F*(1,1053) = 54.175, *p* < 0.05, *η*_p_^2^ = 0.050) with girls showing lower activity times than boys. However, a significant effect of age was only found in device-based PA (*F*(6,1053) = 17.032, *p* < 0.05, *η*_p_^2^ = 0.089) with higher age associated with lower activity. The interaction of sex and age was only significant for self-reported PA (*F*(6,1053) = 2.279, *p* < 0.05, *η*_p_^2^ = 0.013), as boys’ PA levels first decreased, but then increased again after the age of 14, whereas girls’ levels consistently decreased after the age of 13.

Based on the KIDSCREEN-10 index, adolescents showed a mean overall HRQoL of *T* = 52.15 (*SD* = 8.46). Multivariate analyses of variance revealed that females had lower levels of overall HRQoL than males (*F*(1,1423) = 31.215, *p* < 0.05, *η*_p_^2^ = 0.022) and that HRQoL decreased with increasing age (main effect age: *F*(6,1423) = 4.338, *p* < 0.05, *η*_p_^2^ = 0.018; interaction of sex and age: *F*(6,1423) = 4.647, *p* < 0.05, *η*_p_^2^ = 0.019). There were significant main effects of sex on all subscales (*p*-values < 0.05) except for School Environment. Regarding age, all main effects were significant except for Autonomy & Parent Relations and Social Support & Peers (all other *p*’s < 0.05). Also, interactions between sex and age were significant except for Social Support & Peers (all other *p*’s < 0.05). Bonferroni-corrected posthoc tests revealed decreasing HRQoL with increasing age and males having a higher HRQoL than females in almost all subscales except for Social Support & Peers, where females scored higher. Table 1 presents participant characteristics. Means and standard deviations of PA and HRQoL subscales by age and sex can be found in Additional file 1.

**Table 1:** Participant characteristics

	N	Percent (%)	Mean	SD
*Age*	1565		14.37	1.99
*Sex*				
Male	715	45.7		
Female	850	54.3		
*SES*	1545		2.18	0.55
Low	120	7.7		
Medium	1034	66.1		
High	401	25.6		
*PA*				
Self-report	1545		278.62	199.32
Device-based	1068		307.86	134.55
*HRQoL*				
Overall HRQOL (Kidscreen-10 Index)	1432		52.15	8.46
Physical wellbeing	1481		50.1	9.02
Psychological wellbeing	1485		50.95	8.94
Autonomy and parent relations	1474		54.74	0.12
Social support and peers	1479		51.43	8.77
School environment	1455		52.05	7.97

Values for HRQoL reflect calculated T-scores, scaled with a mean of 50 and a standard deviation of 10 for each dimension

### Comparison of relationships between self-reported, device-based measured PA and HRQoL

Overall, significant but weak correlations were found between self-reported and device-based measured PA (*r* = 0.233, *p* < 0.001; *r* = 0.220, *p* < 0.001 when controlled for age and sex). Comparison of correlation coefficients revealed that the correlation coefficients of both PA measures significantly differed in the Social Support & Peers subscale of HRQoL (*z* = 2.452, *p* = 0.008). Self-reported PA correlated more strongly with this subscale (*r* = 0.116, *p* < 0.05) than device-based measured PA (*r* = 0.022, *p* = 0.313). Here, in overall HRQoL and the subscale of School Environment, only self-reported PA significantly correlated with HRQoL but not device-based measured PA. In overall HRQoL and School Environment, however, the difference between correlation coefficients was not significant. For Physical Well-Being and Psychological Well-Being, the correlation coefficient was significant for both self-reported and device-based measured PA, but the difference between them was not significant. Thorough results for the whole sample can be found in Table 2.

**Table 2:** Comparison of correlations between self-reported physical activity, device-based physical activity, and health-related quality of life

	*r*	*z*	*p*	*N*
*Overall HRQoL*		.814	.208	975
PA (self-reported)-HRQoL	.095*			
PA (device-based)-HRQoL	.063			
PA (self-reported)-PA (device-based)	.242*			
*Physical well-being*		1.501	.067	1000
PA (self-reported)-HRQoL	.266*			
PA (device-based)-HRQoL	.210*			
PA (self-reported)- PA (device-based)	.247*			
*Psychological well-being*		.052	.479	1004
PA (self-reported)-HRQoL	.070*			
PA (device-based)-HRQoL	.068*			
PA (self-reported)-PA (device-based)	.248*			
*Autonomy and parent relation*		1.417	.078	997
PA (self-reported)-HRQoL	.060			
PA (device-based)-HRQoL	.005			
PA (self-reported)-PA (device-based)	.250*			
*Social support and peers*		2.425	.008*	1000
PA (self-reported)-HRQoL	.116*			
PA (device-based)-HRQoL	.022			
PA (self-reported)-PA (device-based)	.245*			
*School environment*		.971	.166	986
PA (self-reported)-HRQoL	.065*			
PA (device-based)-HRQoL	.027			
PA (self-reported)-PA (device-based)	.245*			

Levels of significance of Fishers z-tests are Bonferroni-Holm corrected and displayed on a *α* = .05 level (*)

*HRQoL* Health-Related Quality of Life

### Multiple hierarchical regression analysis

Self-reported PA was predictive in all subscales except for Autonomy & Parent Relations. The inclusion of device-based measured PA only increased the amount of explained variance in the Physical Well-Being subscale. For Physical Well-Being, the regression including self-reported PA explained 12.8% (adj. *R*^2^ = 0.128) of variance and this amount of explained variance was significant (*F* = 49.985, *p* < 0.001). After the addition of device-based measured PA to the model, self-reported PA was still predictive of Physical Well-Being (β = 0.233, p < 0.001), as well as device-based measured PA (β = 0.008, p < 0.01). Hence, adding device-based measured PA into the model significantly increased the explained variance by 0.7% (*ΔR*^2^ = 0.007, *ΔF* = 7.603, *p* = 0.006). For thorough results see Table 3. Comparison of Correlations between Self-Reported Physical Activity, Device-based Physical Activity, and Health-Related Quality of Life divided by sex and age can be found in Additional file 2.

**Table 3:** Hierachical linear regression results of self-reported and device-based measures of physical activity (PA) on health-related quality of life (HRQoL) adjusted by age and sex (not displayed)

	*B*	*SE*	*β*	*t*	*p*	*F*	*ad.R*^*2*^	*df1*	*df2*	*p*	*ΔF*	*ΔR*^*2*^	*df1*	*df2*	*p*
*Overall HRQoL*															
Model 1						16.016	.047	3	974	< .001***					
PA (self-reported)	.004	.001	.083	2.687	.007**										
Model 2											.373	.000	1	970	.541
PA (self-reported)	.004	.001	.088	2.719	.007**										
PA (device-based)	− .001	.002	− .021	− .611	.541										
*Physical well-being*															
Model 1						49.985	.128	3	995	< .001**					
PA (self-reported)	.012	.001	.253	8.492	< .001***										
Model 2											7.603	.007	1	995	.006**
PA (self-reported)	.011	.001	.233	7.642	< .001***										
PA (device-based)	.006	.002	.008	2.757	.006**										
*Psychological well-being*															
Model 1						22.111	.059	3	1000	< .001***					
PA (self-reported)	.002	.001	.051	1.653	.099										
Model 2											.505	.000	1	999	.477
PA (self-reported)	.003	.001	.056	1.773	.077										
PA (device-based)	− .002	.002	− .024	− .711	.477										
*Autonomy and Parent Relations*															
Model 1						2.229	.004	3	993	.083					
PA (self-reported)	.002	.001	.054	1.694	.090										
Model 2											.798	.001	1	992	.372
PA (self-reported)	.003	.002	.061	1.857	.064										
PA (device-based)	− .002	.002	− .031	− .893	.372										
*Social Support and Peers*															
Model 1						7.679	.020	3	996	< .001***					
PA (self-reported)	.006	.001	.126	3.996	< .001***										
Model 2											.014	.000	1	995	.905
PA (self-reported)	.006	.001	.125	3.859	< .001***										
PA (device-based)	.000	.002	.004	.120	.905										
*School environment*															
Model 1						13.346	.036	3	982	< .001***					
PA (self-reported)	.003	.001	.067	2.114	.035*										
Model 2											.878	.001	1	981	.349
PA (self-reported)	.003	.001	.074	2.273	.023*										
PA (device-based)	− .002	.002	− .032	− .937	.349										

*p ≤ .05, ** p ≤ .01, *** p ≤ .001

## Discussion

This study aimed to expand upon current evidence on the association of self-reported PA and HRQoL and to extend this knowledge upon the concurrent relation of both, self-reported and device-based measured PA on HRQoL in a sample of adolescents aged 11–17 years living in Germany.

Compared to European norms [37] the current sample was similar regarding HRQoL status. Regarding sex differences, it is noticeable that the overall HRQoL of females decreased throughout maturation whereas boys’ HRQoL stayed almost stable over this period. This finding is in accordance with other studies (e.g. [25]), showing the importance of efforts to sustain the HRQoL levels of adolescent girls. For PA measures, self-reported PA showed distinct patterns in males and females. Whereas PA time decreased in males until the age of 14, it highly increased afterward (from 244 min in 14-year-olds to 339 min in 16-year-olds), with another slight decrease in 17-year-olds. In females, however, PA time decreased from 13 years onwards (from 303 to 238 min in 15-year-olds), with this level being unchanged until 17 years. Again, females seem to be more affected by a decrease of health-related behavior with increasing age, underlining the need for appropriate interventions tailored for females. Device-based measured accelerometer times, however, showed a distinct pattern with almost parallel decreases in activity time in both sexes. Consequently, HRQoL of females may profit even more from enhancing their amounts of PA in order to improve HRQoL or to retain their current level of HRQoL as they grew older [42], especially if their relatively lower PA levels are considered.

### Correlation differences between self-reported and device-based measures of PA and HRQoL

The relation of self-reported and device-based measures of PA is in line with former research which has shown positive correlations between both measures [43, 44]. However, the correlation between both PA measures was rather low, raising doubts on the reliability of both measures. This, however, may be explained by the different nature of both measures, with self-reported PA representing habitual PA, whereas device-based PA is representing actual PA within a specific week. Moreover, the latter is recording all types of PA, whereas the self-reported PA represents sports activities rather than everyday activity including active transport, for example. Regarding correlations to HRQoL, results on self-reported PA are comparable to previous studies [21, 45, 46], revealing an overall relationship to HRQoL and all subscales except for Autonomy & Parent Relations. Device-based PA based on accelerometry, however, was only related to overall HRQoL, Physical Well-Being, and Psychological Well-Being subscale. The difference between correlations of self-report and accelerometer-based activity measures was only significant in the subscale Social Support & Peers. Hence, a larger sample size may be needed to detect further differences in correlations. However, it can be concluded that self-reported PA tends to be more closely related to HRQoL than accelerometry measures.

### The predictive value of self-reported PA

Several studies have shown that self-reported PA is a positive predictor for HRQoL, even in adolescents, which was replicated in the current study. Results on self-reported PA revealed that participant’s overall HRQoL can be increased by one T-value if participants would engage in only 12 min of PA more per day (i.e. 84 min per week). Even if this sounds to be only a slight increase, this increase is quite high as all T-scores are centered at a mean of 50. Authors of the KIDSCREEN Test Manual have suggested setting thresholds for classifying values as being “normal” or “noticeable” by adding or subtracting half a standard deviation to the mean of 50, resulting in a “normal” range between 45 and 55 [37]. Given the assumption that PA influences HRQoL, our results indicate that a child or adolescent which is counted being “noticeable” due to an overall HRQoL score of 44, could heighten this value by 1 point by an increase of only 12 min of being more active per day (84 min per week), then being considered as “normal”. This advice may easily be reached by replacing being driven by walking to school, for instance. However, the cross-sectional design has to be considered when interpreting results, as no assumption about the direction of this relationship can be made.

### Additional value of device-based PA

The inclusion of device-based PA following self-reported PA into the model was only able to explain additional variance in the subscale of Physical Well-Being. This result is contrary to an existing study that found device-based PA to be a better predictor of HRQoL in older adults [20]. These differences in results may be due to different age groups investigated. Moreover, the examined age-span ranging from 11 to 17 years in the current sample may have provoked differences in findings due to large heterogeneity in sociodemographic factors like age and sex. In conclusion, self-reported PA may be a good predictor for HRQoL in children and adolescents living in Germany. Here, individuals’ perceptions may play a role, as perceived habitual (i.e. self-reported) PA may be more closely related to perceived health than actual (i.e. device-based) PA. Therefore, self-reported PA should be considered when examining the relationship between PA and perceived health. However, one reason why self-reported PA might be more closely related to higher HRQoL than device-based measured PA could be that mainly organized leisure-time PA was assessed using the MoMo-PAQ (activities in school or sports clubs), whereas device-based measures captured active transport and other leisure-time activities (including household activities) as well. Today, there is some evidence revealing that the mental health benefits of PA are linked to the life domain where the activity takes place [47].

Moreover, device-based PA measurement is largely advertised as the “state-of-the-art” in PA measurement [22] but measures a different construct: actual PA. In conclusion, self-reported PA and HRQoL are reflective of overall estimations, thus may be more congruent measures concerning time reference, whereas device-based accelerometry focuses on the current week. As it comes to measurements of physical well-being, which can be viewed as a trait more than a state, researchers should account for the relation to device-based measured PA. Moreover, the fact that device-based PA explains additional variance only in the Physical Well-being subscale may be a methodological bias, as both, self-reported PA and HRQoL, were measured in the same way, whereas device-based PA and HRQoL were assessed differently (i.e. device-based vs. self-report). Hence, device-based PA might be only related to the subscale which is contextually most related to its nature [48]. Another conceptual concern to be taken into account is that both measures assessed different aspects of the same construct. Even if both measured PA within one week, self-reported measurement asked for PA within a typical week, whereas device-based measurement included exactly the week of measurement. The MoMo-PAQ measures habitual PA whereas the accelerometer measures current PA [26]. This could be one reason why the correlation between device-based and self-reported measured PA is rather low [17]. Both indicators, therefore, are not congruent and their correlations have to be viewed carefully.

### Strengths and limitations

To the best of our knowledge, this is the first study to examine the association of both, self-reported and device-based measurements of PA with HRQoL in a nationwide children and adolescent sample in Germany. The use of a multidimensional, validated measure of HRQoL facilitated to account for national differences and made results comparable to (European) norms. Moreover, accounting for different subscales of HRQoL provided detailed insights into a variety of aspects forming HRQoL and to investigate differential effects of both kinds of activity measurements (i.e. habitual versus actual PA) as well as age and sex differences.

However, some limitations also need to be considered. First, results are difficult to compare to studies from other countries as well as to studies using different age groups (i.e. children vs. adults). Comparing the current sample with data of children may increase the risk of missing factors that are important to adolescents in other ways than to children, such as developing needs for intimate relations, sexuality, and becoming more autonomous towards parents. Moreover, studies assessing the relationship of PA and HRQoL in children and adolescents usually used restricted samples regarding age (e.g. children aged 9–11 years; [46, 49]), not allowing a comparison to the current sample covering an age range of 7 years. On the other hand, comparing adolescents to adults might cause an overlooking of aspects such as striving for autonomy, the importance of peers, the developmental aspects of both intimacy and sexuality, and the not yet fully developed ability to take responsibility for one's actions.

Second, self-reports are more likely to be influenced by social desirability than device-based measures and therefore need to be interpreted carefully as also stated in a recent meta-analysis [21]. Furthermore, it can be assumed that higher correlations between self-reported PA and HRQoL as self-report measure might be due to a method effect. Therefore, the difference in the correlations should be interpreted with caution. However, accelerometers also have methodological issues that have to be considered while interpreting findings. First, it should be noticed that non-locomotive movements of the body (e. g. cycling) cannot be recorded by accelerometers, which is critical in terms of validity [17, 50]. Further, accelerometers are only able to gain reliable data if they are worn regularly. As device-based data, however, revealed higher PA than self-reported measures, a lack of wear-time can be denied. The higher values in the accelerometer measured PA could come from the fact that in the self-reported data "only" a sports index is used to calculate the minutes of moderate-to-vigorous PA in a week. This PA that the participants generate in their everyday life besides sports activities (for example by running up the stairs), is only recorded with the accelerometers and is missing in the sports index. Even as inclusion criteria of the current study were chosen according to recent guidelines [51] and median wear-time was high, interindividual wear-time differences still have to be kept in mind, as total PA time differs concerning wear-time (i.e. higher wear-time usually elicits higher PA time). Some device-based data (which should be representative for a whole week) only represents five wearing-days, leaving the chance of underestimation of total activity time. Though, as both, self-reported and device-based measures of PA were correlated to some extent, this issue is not a cause for concern. However, the rather low correlation between both measurement methods may rather be interpreted in terms of different constructs measured: whereas self-reported PA measures habitual PA, device-based PA measured the current PA within the specific examination week. Therefore, the results of both methods may differ due to environmental reasons.

Third, the cross-sectional comparison does not allow for causal conclusions. Future research needs to examine the question about the direction of the PA-HRQoL relationship using longitudinal data.

### Future directions and conclusion

Results from the current investigation point to a self-reported PA being a more important predictor for a comprehensive investigation of PA and HRQoL relations. However, as both kinds of PA measures showed a weak but significant correlation, future studies should aim on developing more reliable measures of PA to be used in future studies. Future studies should also aim at investigating if device-based measures of PA are more sensitive for physical quality-of-life (as well as biometric and/or biological) outcomes compared to self-reported PA measures and therefore if both measures represent different traits. For this purpose, multitrait-multimethod-analysis may be an appropriate approach. Multiple procedures (i.e. self-report vs. accelerometry) could be used to measure multiple theoretical constructs to determine whether the measurements of each trait derived by multiple methods are concordant [52]. Moreover, it should be replicated and confirmed if self-reported PA measures are more sensitive for social-oriented outcomes, and if self-reports correlate to a higher extent to self-reports than to device-based measures. Therefore, future studies should aim for convergent assessment of self-reported and device-based actual PA as well as an assessment of different self-reported and device-based measured health constructs (e.g. calorimetry vs. self-reported calorie intake, cortisol vs. self-reported stress levels, measured BMI vs. self-reported BMI, etc.).

Hence, findings point to the importance of PA as a possible approach to enhance HRQoL. As HRQOL is known to be a description of holistic health [53] and to be highly correlated to mortality in higher ages [54], future studies should investigate the direct effect of PA on HRQoL and health in a longitudinal approach to account for the causality of effects. These findings can lay a foundation for public health as PA interventions can be individually designed to maintain high HRQoL standards from youth onwards until higher ages.

Regarding time and cost-effectiveness, researchers conducting future studies on self-reported measured constructs like HRQoL can be advised to reflect if they want to use self-reported or device-based measured PA when examining the effects on HRQoL. Examinations including accelerometry should use systems that can stay attached to the participant’s body for the complete measurement period to avoid differences in wear-time registration, making it easier to interpret results if they parallel self-reported measures and can be reported in “minutes per day or per week”. Future studies should also account for dose–response-relations of self-reported and device-based measured PA and HRQoL in children and adolescents [54].

Taken together, results showed that self-reported and device-based measured PA were moderately correlated regarding overall HRQoL and all subscales. Further, results revealed that self-reported PA was a significant predictor for overall HRQoL and all subscales except for Autonomy & Parent Relations. However, there was only an additional effect of device-based PA on HRQoL in Physical Well-Being. In conclusion, self-reported measured PA explains more variance when regressed on HRQoL than device-based measured PA, even though the measures showed a low correlation. From a public health perspective, a better understanding of how healthy lifestyles, such as uptake of PA, can be related to HRQoL might help to inform policy intended to incentivize PA in the general population [15].

## Supplementary information

**Additional file 1.** Means and standard deviations of total physical activity (PA; minutes per week) and all health-related quality of life (HRQoL)subscales differentiated by sex and age groups.

**Additional file 2.** Comparison of Correlations between Self-Reported Physical Activity, Device-based Physical Activity, and Health-Related Quality of Life differentiated by sex and age groups. 2a) Comparison of Correlations between Self-Reported Physical Activity, Device-based Physical Activity, and Health-Related Quality of Life in females. 2b) Comparison of Correlations between Self-Reported Physical Activity, Device-based Physical Activity, and Health-Related Quality of Life in males. 2c) Comparison of Correlations between Self-Reported Physical Activity, Device-based Physical Activity, and Health-Related Quality of Life in young participants aged 11 to 14 years. 2d) Comparison of Correlations between Self-Reported Physical Activity, Device-based Physical Activity, and Health-Related Quality of Life older participants aged 15 to 17 years.

## Data Availability

The datasets used and/or analysed during the current study are available from the corresponding author on reasonable request.

## Abbreviations

PA: Physical activity
HRQoL: Health-related quality of life
MoMo-PAQ: MoMo Physical Activity Questionnaire
SES: Socioeconomic status
