# Supplementary material for: The relationship of self-reported and device-based measures of physical activity and health-related quality of life in adolescents
Source: Health Qual Life Outcomes. 2021 Mar 1;19:67. doi: 10.1186/s12955-021-01682-3 (PMC7923541; doi:10.1186/s12955-021-01682-3)
Supplement: Supplementary file 1 — Additional file 1. Means and standard deviations of total physical activity (PA; minutes per week) and all health-related quality of life (HRQoL)subscales differentiated by sex and age groups. [file 12955_2021_1682_MOESM1_ESM.docx]

*Additional File 1.* Means and standard deviations of total physical activity (PA; minutes per week) and all health-related quality of life (HRQoL) subscales differentiated by sex and age groups

|  |  |  | male | | |  | female | | |  |  |  |  |
| --- | --- | --- | --- | --- | --- | --- | --- | --- | --- | --- | --- | --- | --- |
|  |  |  | n | Mean | SD |  | n | Mean | SD |  | *T* | *p* |  |
| **11-year old** | |  |  |  |  |  |  |  |  |  |  |  |  |
|  | **HRQoL** | |  |  |  |  |  |  |  |  |  |  |  |
|  |  | Overall HRQoL (Kidscreen-10 Index) | 80 | 54.34 | 8.70 |  | 92 | 54.26 | 9.71 |  | -0.790 | 0.431 |  |
|  |  | Physical Wellbeing | 83 | 53.98 | 8.77 |  | 93 | 54.77 | 8.99 |  | -0.962 | 0.338 |  |
|  |  | Psychological Wellbeing | 83 | 53.10 | 8.11 |  | 93 | 52.82 | 9.11 |  | 0.179 | 0.858 |  |
|  |  | Autonomy & Parent Relations | 83 | 54.74 | 9.87 |  | 92 | 54.11 | 9.43 |  | -0.607 | 0.545 |  |
|  |  | Social Support & Peers | 83 | 50.72 | 7.37 |  | 93 | 52.22 | 8.92 |  | -1.483 | 0.141 |  |
|  |  | School Environment | 83 | 54.52 | 8.47 |  | 93 | 55.71 | 9.13 |  | -1.578 | 0.117 |  |
|  | **PA** |  |  |  |  |  |  |  |  |  |  |  |  |
|  |  | Self-reported | 112 | 314.70 | 183.92 |  | 118 | 263.85 | 147.52 |  | 1.426 | 0.156 |  |
|  |  | Device-based | 73 | 445.96 | 176.28 |  | 92 | 350.76 | 110.30 |  | 3.255 | 0.002 | * |
| **12-year old** | |  |  |  |  |  |  |  |  |  |  |  |  |
|  | **HRQoL** | |  |  |  |  |  |  |  |  |  |  |  |
|  |  | Overall HRQoL (Kidscreen-10 Index) | 100 | 53.78 | 8.48 |  | 126 | 53.53 | 8.89 |  | 0.730 | 0.467 |  |
|  |  | Physical Wellbeing | 103 | 52.47 | 8.80 |  | 130 | 51.04 | 7.99 |  | 1.120 | 0.264 |  |
|  |  | Psychological Wellbeing | 104 | 53.16 | 8.19 |  | 132 | 52.07 | 8.17 |  | 1.596 | 0.113 |  |
|  |  | Autonomy & Parent Relations | 103 | 54.40 | 9.29 |  | 131 | 55.44 | 9.55 |  | 0.155 | 0.877 |  |
|  |  | Social Support & Peers | 103 | 50.70 | 9.13 |  | 131 | 51.70 | 9.20 |  | -0.757 | 0.450 |  |
|  |  | School Environment | 103 | 53.64 | 8.06 |  | 128 | 53.19 | 7.45 |  | 1.224 | 0.223 |  |
|  | **PA** |  |  |  |  |  |  |  |  |  |  |  |  |
|  |  | Self-reported | 108 | 311.92 | 174.90 |  | 135 | 263.44 | 215.99 |  | 1.841 | 0.067 |  |
|  |  | Device-based | 79 | 347.85 | 119.86 |  | 93 | 297.32 | 127.08 |  | 2.765 | 0.006 | * |
| **13-year old** | |  |  |  |  |  |  |  |  |  |  |  |  |
|  | **HRQoL** | |  |  |  |  |  |  |  |  |  |  |  |
|  |  | Overall HRQoL (Kidscreen-10 Index) | 98 | 52.56 | 7.90 |  | 119 | 52.13 | 7.38 |  | -0.102 | 0.919 |  |
|  |  | Physical Wellbeing | 103 | 51.15 | 8.28 |  | 122 | 50.39 | 8.77 |  | 0.002 | 0.998 |  |
|  |  | Psychological Wellbeing | 103 | 52.23 | 7.83 |  | 122 | 50.99 | 8.06 |  | 0.561 | 0.576 |  |
|  |  | Autonomy & Parent Relations | 103 | 54.60 | 8.36 |  | 120 | 54.96 | 9.85 |  | -0.558 | 0.578 |  |
|  |  | Social Support & Peers | 102 | 49.71 | 8.57 |  | 122 | 53.44 | 9.64 |  | -2.523 | 0.013 |  |
|  |  | School Environment | 101 | 50.44 | 7.86 |  | 121 | 51.83 | 7.58 |  | -1.726 | 0.087 |  |
|  | **PA** |  |  |  |  |  |  |  |  |  |  |  |  |
|  |  | Self-reported | 103 | 296.51 | 186.57 |  | 121 | 291.31 | 213.38 |  | -0.459 | 0.647 |  |
|  |  | Device-based | 66 | 331.36 | 134.68 |  | 92 | 280.90 | 125.94 |  | 2.466 | 0.015 |  |
| **14-year old** | |  |  |  |  |  |  |  |  |  |  |  |  |
|  | **HRQoL** | |  |  |  |  |  |  |  |  |  |  |  |
|  |  | Overall HRQoL (Kidscreen-10 Index) | 107 | 52.88 | 7.38 |  | 100 | 51.46 | 7.93 |  | 0.274 | 0.784 |  |
|  |  | Physical Wellbeing | 107 | 50.52 | 9.18 |  | 103 | 48.12 | 8.66 |  | 0.629 | 0.530 |  |
|  |  | Psychological Wellbeing | 108 | 52.69 | 7.97 |  | 103 | 49.65 | 9.31 |  | 1.753 | 0.082 |  |
|  |  | Autonomy & Parent Relations | 107 | 54.90 | 7.62 |  | 103 | 55.93 | 9.60 |  | -0.948 | 0.345 |  |
|  |  | Social Support & Peers | 108 | 50.27 | 7.24 |  | 103 | 52.27 | 8.58 |  | -1.566 | 0.120 |  |
|  |  | School Environment | 107 | 51.57 | 7.94 |  | 102 | 52.31 | 7.82 |  | -0.479 | 0.633 |  |
|  | **PA** |  |  |  |  |  |  |  |  |  |  |  |  |
|  |  | Self-reported | 106 | 255.68 | 168.18 |  | 105 | 257.93 | 203.45 |  | -0.517 | 0.606 |  |
|  |  | Device-based | 67 | 297.55 | 128.00 |  | 73 | 283.01 | 106.78 |  | 0.440 | 0.661 |  |
| **15-year old** | |  |  |  |  |  |  |  |  |  |  |  |  |
|  | **HRQoL** | |  |  |  |  |  |  |  |  |  |  |  |
|  |  | Overall HRQoL (Kidscreen-10 Index) | 98 | 53.29 | 9.43 |  | 130 | 49.20 | 8.32 |  | 2.466 | 0.015 |  |
|  |  | Physical Wellbeing | 100 | 50.78 | 9.48 |  | 133 | 46.61 | 8.28 |  | 2.741 | 0.007 | * |
|  |  | Psychological Wellbeing | 100 | 52.39 | 9.70 |  | 133 | 47.69 | 9.83 |  | 2.295 | 0.023 |  |
|  |  | Autonomy & Parent Relations | 100 | 56.15 | 9.02 |  | 132 | 53.50 | 8.63 |  | 1.993 | 0.048 |  |
|  |  | Social Support & Peers | 100 | 51.42 | 8.73 |  | 133 | 51.38 | 9.57 |  | 0.449 | 0.654 |  |
|  |  | School Environment | 99 | 51.45 | 8.70 |  | 131 | 50.40 | 7.86 |  | 0.769 | 0.443 |  |
|  | **PA** |  |  |  |  |  |  |  |  |  |  |  |  |
|  |  | Self-reported | 101 | 319.80 | 229.27 |  | 134 | 234.99 | 171.96 |  | 1.985 | 0.049 |  |
|  |  | Device-based | 72 | 314.21 | 126.09 |  | 93 | 256.17 | 109.06 |  | 3.018 | 0.003 | * |
| **16-year old** | |  |  |  |  |  |  |  |  |  |  |  |  |
|  | **HRQoL** | |  |  |  |  |  |  |  |  |  |  |  |
|  |  | Overall HRQoL (Kidscreen-10 Index) | 97 | 53.31 | 7.27 |  | 121 | 49.87 | 7.60 |  | 2.884 | 0.005 | * |
|  |  | Physical Wellbeing | 99 | 51.44 | 8.95 |  | 130 | 46.93 | 8.46 |  | 2.682 | 0.008 | * |
|  |  | Psychological Wellbeing | 99 | 52.78 | 9.05 |  | 130 | 48.63 | 8.78 |  | 3.805 | <0.001 | * |
|  |  | Autonomy & Parent Relations | 98 | 55.56 | 8.72 |  | 128 | 53.80 | 9.19 |  | 1.518 | 0.131 |  |
|  |  | Social Support & Peers | 99 | 50.65 | 7.78 |  | 128 | 51.85 | 8.48 |  | -1.900 | 0.060 |  |
|  |  | School Environment | 99 | 51.18 | 6.78 |  | 122 | 51.49 | 7.12 |  | -1.065 | 0.289 |  |
|  | **PA** |  |  |  |  |  |  |  |  |  |  |  |  |
|  |  | Self-reported | 98 | 328.19 | 224.78 |  | 131 | 253.36 | 203.97 |  | 2.137 | 0.034 |  |
|  |  | Device-based | 58 | 306.22 | 121.43 |  | 90 | 258.24 | 108.22 |  | 2.577 | 0.011 |  |
| **17-year old** | |  |  |  |  |  |  |  |  |  |  |  |  |
|  | **HRQoL** | |  |  |  |  |  |  |  |  |  |  |  |
|  |  | Overall HRQoL (Kidscreen-10 Index) | 70 | 54.37 | 9.56 |  | 94 | 47.10 | 6.84 |  | 3.737 | <0.001 | * |
|  |  | Physical Wellbeing | 78 | 51.03 | 8.81 |  | 97 | 44.86 | 8.13 |  | 4.082 | <0.001 | * |
|  |  | Psychological Wellbeing | 78 | 51.23 | 8.89 |  | 97 | 45.46 | 8.12 |  | 2.433 | 0.017 |  |
|  |  | Autonomy & Parent Relations | 77 | 57.61 | 9.72 |  | 97 | 51.52 | 7.89 |  | 3.060 | 0.003 | * |
|  |  | Social Support & Peers | 77 | 52.40 | 8.24 |  | 97 | 50.94 | 9.38 |  | 0.560 | 0.576 |  |
|  |  | School Environment | 72 | 52.98 | 7.83 |  | 94 | 49.17 | 7.28 |  | 0.972 | 0.333 |  |
|  | **PA** |  |  |  |  |  |  |  |  |  |  |  |  |
|  |  | Self-reported | 76 | 307.94 | 248.27 |  | 97 | 230.13 | 193.27 |  | 2.290 | 0.024 |  |
|  |  | Device-based | 50 | 323.00 | 170.14 |  | 70 | 241.94 | 98.80 |  | 2.667 | 0.010 | * |

*Note.* Values for HRQoL reflect calculated T-scores, scaled with a mean of 50 and a standard deviation of 10 for each dimension. Levels of significance are Bonferroni-Holm corrected and displayed on a *α*=.05 level (*).
