# Supplementary material for: The relationship of self-reported and device-based measures of physical activity and health-related quality of life in adolescents
Source: Health Qual Life Outcomes. 2021 Mar 1;19:67. doi: 10.1186/s12955-021-01682-3 (PMC7923541; doi:10.1186/s12955-021-01682-3)
Supplement: Supplementary file 2 — Additional file 2. Comparison of Correlations between Self-Reported Physical Activity, Device-based Physical Activity, and Health-Related Quality of Life differentiated by sex and age groups. 2a) Comparison of Correlations between Self-Reported Physical Activity, Device-based Physical Activity, and Health-Related Quality of Life in females. 2b) Comparison of Correlations between Self-Reported Physical Activity, Device-based Physical Activity, and Health-Related Quality of Life in males. 2c) Comparison of Correlations between Self-Reported Physical Activity, Device-based Physical Activity, and Health-Related Quality of Life in young participants aged 11 to 14 years. 2d) Comparison of Correlations between Self-Reported Physical Activity, Device-based Physical Activity, and Health-Related Quality of Life older participants aged 15 to 17 years. [file 12955_2021_1682_MOESM2_ESM.docx]

*Additional File 2.* Comparison of Correlations between Self-Reported Physical Activity, Device-based Physical Activity, and Health-Related Quality of Life differentiated by sex and age groups.

*2a.* Comparison of Correlations between Self-Reported Physical Activity, Device-based Physical Activity, and Health-Related Quality of Life in females.

|  | *r* | *z* | *p* | *N* |
| --- | --- | --- | --- | --- |
| **Overall HRQoL** |  | .113 | .455 | 553 |
| PA (Self-reported) - HRQoL | .121* |  |  |  |
| PA (Device-based) - HRQoL | .115* |  |  |  |
| PA (Self-reported) - PA (Device-based) | .209* |  |  |  |
| **Physical Well-Being** |  | 1.313 | .095 | 569 |
| PA (Self-reported) - HRQoL | .290* |  |  |  |
| PA (Device-based) - HRQoL | .224* |  |  |  |
| PA (Self-reported) - PA (Device-based) | .212* |  |  |  |
| **Psychological Well-Being** |  | -0.248 | .402 | 571 |
| PA (Self-reported) - HRQoL | .079 |  |  |  |
| PA (Device-based) - HRQoL | .092* |  |  |  |
| PA (Self-reported) - PA (Device-based) | .212* |  |  |  |
| **Autonomy & Parent Relation** |  | .399 | .345 | 566 |
| PA (Self-reported) - HRQoL | .084* |  |  |  |
| PA (Device-based) - HRQoL | .063 |  |  |  |
| PA (Self-reported) - PA (Device-based) | .215* |  |  |  |
| **Social Support & Peers** |  | 2.377 | .009* | 568 |
| PA (Self-reported) - HRQoL | .160* |  |  |  |
| PA (Device-based) - HRQoL | .035 |  |  |  |
| PA (Self-reported) - PA (Device-based) | .206* |  |  |  |
| **School Environment** |  | .847 | .199 | 558 |
| PA (Self-reported) - HRQoL | .100* |  |  |  |
| PA (Device-based) - HRQoL | .055 |  |  |  |
| PA (Self-reported) - PA (Device-based) | .209* |  |  |  |

*Note.* Levels of significance of Fishers z-tests are Bonferroni-Holm corrected and displayed on a *α*=.05 level (*). HRQoL=Health-Related Quality of Life.

*2b.* Comparison of Correlations between Self-Reported Physical Activity, Device-based Physical Activity, and Health-Related Quality of Life in males.

|  | *r* | *z* | *p* | *N* |
| --- | --- | --- | --- | --- |
| **Overall HRQoL** |  | 1.235 | .108 | 422 |
| PA (Self-reported) - HRQoL | .034 |  |  |  |
| PA (Device-based) - HRQoL | -.040 |  |  |  |
| PA (Self-reported) - PA (Device-based) | .247* |  |  |  |
| **Physical Well-Being** |  | .936 | .175 | 431 |
| PA (Self-reported) - HRQoL | .211* |  |  |  |
| PA (Device-based) - HRQoL | .157* |  |  |  |
| PA (Self-reported) - PA (Device-based) | .252* |  |  |  |
| **Psychological Well-Being** |  | .730 | .233 | 433 |
| PA (Self-reported) - HRQoL | .018 |  |  |  |
| PA (Device-based) - HRQoL | -.025 |  |  |  |
| PA (Self-reported) - PA (Device-based) | .253* |  |  |  |
| **Autonomy & Parent Relation** |  | 1.627 | .052 | 431 |
| PA (Self-reported) - HRQoL | .013 |  |  |  |
| PA (Device-based) - HRQoL | -.083 |  |  |  |
| PA (Self-reported) - PA (Device-based) | .253* |  |  |  |
| **Social Support & Peers** |  | .509 | .305 | 432 |
| PA (Self-reported) - HRQoL | .076 |  |  |  |
| PA (Device-based) - HRQoL | .046 |  |  |  |
| PA (Self-reported) - PA (Device-based) | .252* |  |  |  |
| **School Environment** |  | .320 | .375 | 428 |
| PA (Self-reported) - HRQoL | .026 |  |  |  |
| PA (Device-based) - HRQoL | .007 |  |  |  |
| PA (Self-reported) - PA (Device-based) | .250* |  |  |  |

*Note.* Levels of significance of Fishers z-tests are Bonferroni-Holm corrected and displayed on a *α*=.05 level (*). HRQoL=Health-Related Quality of Life.

*2c.* Comparison of Correlations between Self-Reported Physical Activity, Device-based Physical Activity, and Health-Related Quality of Life in young participants aged 11 to 14 years.

|  | *r* | *z* | *p* | *N* |
| --- | --- | --- | --- | --- |
| **Overall HRQoL** |  | 1.105 | .135 | 565 |
| PA (Self-reported) - HRQoL | .113* |  |  |  |
| PA (Device-based) - HRQoL | .057 |  |  |  |
| PA (Self-reported) - PA (Device-based) | .271* |  |  |  |
| **Physical Well-Being** |  | -.062 | .475 | 576 |
| PA (Self-reported) - HRQoL | .220* |  |  |  |
| PA (Device-based) - HRQoL | .223* |  |  |  |
| PA (Self-reported) - PA (Device-based) | .270* |  |  |  |
| **Psychological Well-Being** |  | 1.258 | .104 | 580 |
| PA (Self-reported) - HRQoL | .091* |  |  |  |
| PA (Device-based) - HRQoL | .028 |  |  |  |
| PA (Self-reported) - PA (Device-based) | .272* |  |  |  |
| **Autonomy & Parent Relation** |  | .832 | .203 | 574 |
| PA (Self-reported) - HRQoL | .051 |  |  |  |
| PA (Device-based) - HRQoL | .009 |  |  |  |
| PA (Self-reported) - PA (Device-based) | .272* |  |  |  |
| **Social Support & Peers** |  | 2.310 | .010* | 577 |
| PA (Self-reported) - HRQoL | .139* |  |  |  |
| PA (Device-based) - HRQoL | .023 |  |  |  |
| PA (Self-reported) - PA (Device-based) | .268* |  |  |  |
| **School Environment** |  | 1.150 | .125 | 572 |
| PA (Self-reported) - HRQoL | .068 |  |  |  |
| PA (Device-based) - HRQoL | .010 |  |  |  |
| PA (Self-reported) - PA (Device-based) | .274* |  |  |  |

*Note.* Levels of significance of Fishers z-tests are Bonferroni-Holm corrected and displayed on a *α*=.05 level (*). HRQoL=Health-Related Quality of Life.

*2d.* Comparison of Correlations between Self-Reported Physical Activity, Device-based Physical Activity, and Health-Related Quality of Life older participants aged 15 to 17 years.

|  | *r* | *z* | *p* | *N* |
| --- | --- | --- | --- | --- |
| **Overall HRQoL** |  | .951 | .171 | 410 |
| PA (Self-reported) - HRQoL | .073 |  |  |  |
| PA (Device-based) - HRQoL | .014 |  |  |  |
| PA (Self-reported) - PA (Device-based) | .214* |  |  |  |
| **Physical Well-Being** |  | 3.390 | <.001* | 424 |
| PA (Self-reported) - HRQoL | .327* |  |  |  |
| PA (Device-based) - HRQoL | .130* |  |  |  |
| PA (Self-reported) - PA (Device-based) | .227* |  |  |  |
| **Psychological Well-Being** |  | -.215 | .415 | 424 |
| PA (Self-reported) - HRQoL | .046 |  |  |  |
| PA (Device-based) - HRQoL | .059 |  |  |  |
| PA (Self-reported) - PA (Device-based) | .227* |  |  |  |
| **Autonomy & Parent Relation** |  | 1.423 | .077 | 423 |
| PA (Self-reported) - HRQoL | .071 |  |  |  |
| PA (Device-based) - HRQoL | -.015 |  |  |  |
| PA (Self-reported) - PA (Device-based) | .231* |  |  |  |
| **Social Support & Peers** |  | 1.221 | .111 | 423 |
| PA (Self-reported) - HRQoL | .088 |  |  |  |
| PA (Device-based) - HRQoL | .014 |  |  |  |
| PA (Self-reported) - PA (Device-based) | .225* |  |  |  |
| **School Environment** |  | 1.117 | .132 | 414 |
| PA (Self-reported) - HRQoL | .063 |  |  |  |
| PA (Device-based) - HRQoL | -.006 |  |  |  |
| PA (Self-reported) - PA (Device-based) | .215* |  |  |  |

*Note.* Levels of significance of Fishers z-tests are Bonferroni-Holm corrected and displayed on a *α*=.05 level (*). HRQoL=Health-Related Quality of Life.
